# Associations between riffle development and aquatic biota following lowhead dam removal

**DOI:** 10.1007/s10661-018-6716-1

**Published:** 2018-05-10

**Authors:** Danielle R. Cook, S. Mažeika P. Sullivan

**Affiliations:** Schiermeier Olentangy River Wetland Research Park, School of Environment and Natural Resources, The Ohio State University, Columbus, OH 43202 USA

**Keywords:** Aquatic biodiversity, Dam removal, Darter, Hydrogeomorphology, River restoration

## Abstract

Dam removal is an increasingly common river restoration option, yet some of the mechanisms leading to ecological changes remain unquantified. We assessed relationships between riffle structure and benthic macroinvertebrate and fish assemblages 2 years after a lowhead dam removal in Ohio, USA. Hydrogeomorphic, water-chemistry, and biotic surveys were conducted at seven study riffles at six time intervals from spring 2014 through summer 2015. The density and diversity of macroinvertebrates and fish were significantly different over time, largely as a function of season (lowest densities in early spring, greatest in summer). Macroinvertebrate, but not fish, assemblage composition was different by time but not riffle. Although hydrogeomorphic characteristics (e.g., streamflow velocity, substrate size) were linked to shifts in macroinvertebrates and fish, chemical water-quality parameters (e.g., dissolved oxygen, nutrient concentrations) were also implicated as potential biotic drivers. Our results indicate that riffle habitat development can be an important mechanism related to restoring sensitive species and biological diversity following dam removal.

**Electronic supplementary material:**

The online version of this article (10.1007/s10661-018-6716-1) contains supplementary material, which is available to authorized users.

## Introduction

Riffles—shallow sections of streams or rivers with rapid current—are important habitat units for benthic macroinvertebrate and fish assemblages (Kessler and Thorp 1993; Kessler et al. 1995; Heino et al. 2004). Riffles increase water turbulence and oxygen concentration and provide important microhabitat variability in depth, velocity, and substrate for aquatic macroinvertebrates (Statzner et al. 1988; Merritt et al. 2008). The high substrate heterogeneity typical of riffles (Gordon et al. 2004) also allows multiple fish species to coexist through spatial partitioning of habitat (Kessler and Thorp 1993). Likewise, riffles can support higher densities of benthic macroinvertebrates than pool habitats and are important areas of food production for insectivorous fishes (Scullion et al. 1982; Gordon et al. 2004).

Even relatively small river infrastructure, such as weirs and run-of-river, lowhead dams (< 7.6 m in height) impound water and can cause a general flattening of the channel and fining of streambed substrate, leading to a loss of riffles and gravel substrates upstream of the structure (Doyle et al. 2005; Salant et al. 2012). However, in recent years, increasing numbers of dams have been removed owing to failing infrastructure, impounded sediment, danger posed to humans, or general lack of utility (Bednarek 2001; American Rivers 2015).

Channel responses to dam removal vary considerably depending on channel characteristics and sediment regimes (Doyle et al. 2003; Wildman and MacBroom 2005), and stem from complex adjustment processes between channel aggradation and degradation (Bushaw-Newton et al. 2002; Cooper 2013). Responses to dam removal can be immediate to longer term (Hart et al. 2002; Doyle et al. 2003), although increasing evidence suggests that many river responses can occur within months, not years (Grant and Lewis 2015). Maloney et al. (2008) found that bed particle size both above and below the dam site increased within 1 year following the removal of a lowhead dam (105 m wide and 1.7 m in height) on the Fox River, Illinois, USA. This finding runs counter to other studies that have documented a short-term decrease in particle size at downstream reaches following dam removal (e.g., Thomson et al. 2005). In some gravel-bed rivers, the transport rate of sediment exceeds the sediment- supply rate; thus, all but the coarsest material was rapidly removed in the former impounded areas 5 years following four lowhead dam removals in Connecticut, USA (Wildman and MacBroom 2005). Finer-grained sediments flushed downstream can expose riffles in the former impoundment (Egan 2001). Conversely, courser-grained riffles can be buried by finer-grained sediment being transported to downstream reaches following dam removal (Pizzuto 2002). Marked changes in channel gradient can also occur via the development of knick points (Schumm et al. 1984; Doyle et al. 2003).

The redevelopment of riffles following dam removal may be an important factor related to the effects of dam removal on riverine biotic communities (e.g., Sullivan and Manning 2017). For instance, an initial increase in macroinvertebrate abundance has been observed upstream of previous dam sites (Bushaw-Newton et al. 2002; Maloney et al. 2008) in contrast to declines in abundance downstream (Thomson et al. 2005). Maloney et al. (2008) found that the relative abundance of Ephemeroptera, Plecoptera, and Trichoptera (EPT) increased—largely due to increased hydropsychid caddisfly abundance—in the formerly impounded area following removal of a lowhead dam. Cooper (2013) observed that while the total number of macroinvertebrates increased, there was no significant difference in the number of EPT families when comparing pre-dam to post-dam years on the 4th-order Salmon River in Quebec, Canada. Macroinvertebrate community responses can be both rapid (e.g., 2 weeks; Orr et al. 2008), as well as occur over longer time scales (e.g., 3.5 years; Renöfalt et al. 2013).

Several studies have shown that fish species richness and diversity tend to increase upstream of previous dam locations (e.g., Catalano et al. 2007; Ross et al. 2001), returning to lotic-type communities (Bushaw-Newton et al. 2002). Conversely, downstream fish assemblages have been shown to decline in species richness, abundance, and diversity shortly following dam removal (Catalano et al. 2007; Gardner et al. 2013). In particular, the potential redevelopment of riffles following dam removal may be of particular benefit to aquatic biota. Bushaw-Newton et al. (2002), for instance, found that riffle fish species (e.g., Tessellated Darter [*Etheostoma olmstedi*], Shield Darter [*Percina peltata*], and Hog Sucker [*Hypentelium nigricans*]) moved into newly formed riffles upstream of a former impoundment 1 year after dam removal in a 4th-order stream in southeastern Pennsylvania, USA.

As dam removal and subsequent restoration projects become more common (Pohl 2002; O’Connor et al. 2015), understanding how rivers change following dam removal is of increasing importance for both science and management. In this study, we monitored how post-dam removal riffle development influenced aquatic biota. This was not a before-after study; rather, we assessed the associations between riffle structure and benthic macroinvertebrate and fish assemblages 1.5–3 years following the removal of a lowhead dam on the 5th-order Olentangy River of central Ohio, USA. We predicted that riffles that developed in the previously impounded section of the river (via both in-channel restoration activities and natural geomorphic processes) would be characterized by increased mean sediment particle size, streambed slope, and streamflow variability over time with concomitant increases in the density and diversity of both benthic macroinvertebrate and fish assemblages (although seasonal variation was expected; e.g., Sullivan and Manning 2017). We also hypothesized that dissimilarities in species composition and mean abundance of macroinvertebrates and fish above and below the previous dam would decrease over time. Although our focus was on hydrogeomorphic-biotic relationships of riffles following dam removal, we also anticipated that chemical water quality would contribute to explaining patterns in macroinvertebrate and fish assemblages, owing to the importance of water chemistry to both fishes and macroinvertebrates (Wynes and Wissing 1981; Rosenberg and Resh 1993; Hering et al. 2006).

## Materials and methods

### Study system and experimental design

**Fig. 1:**
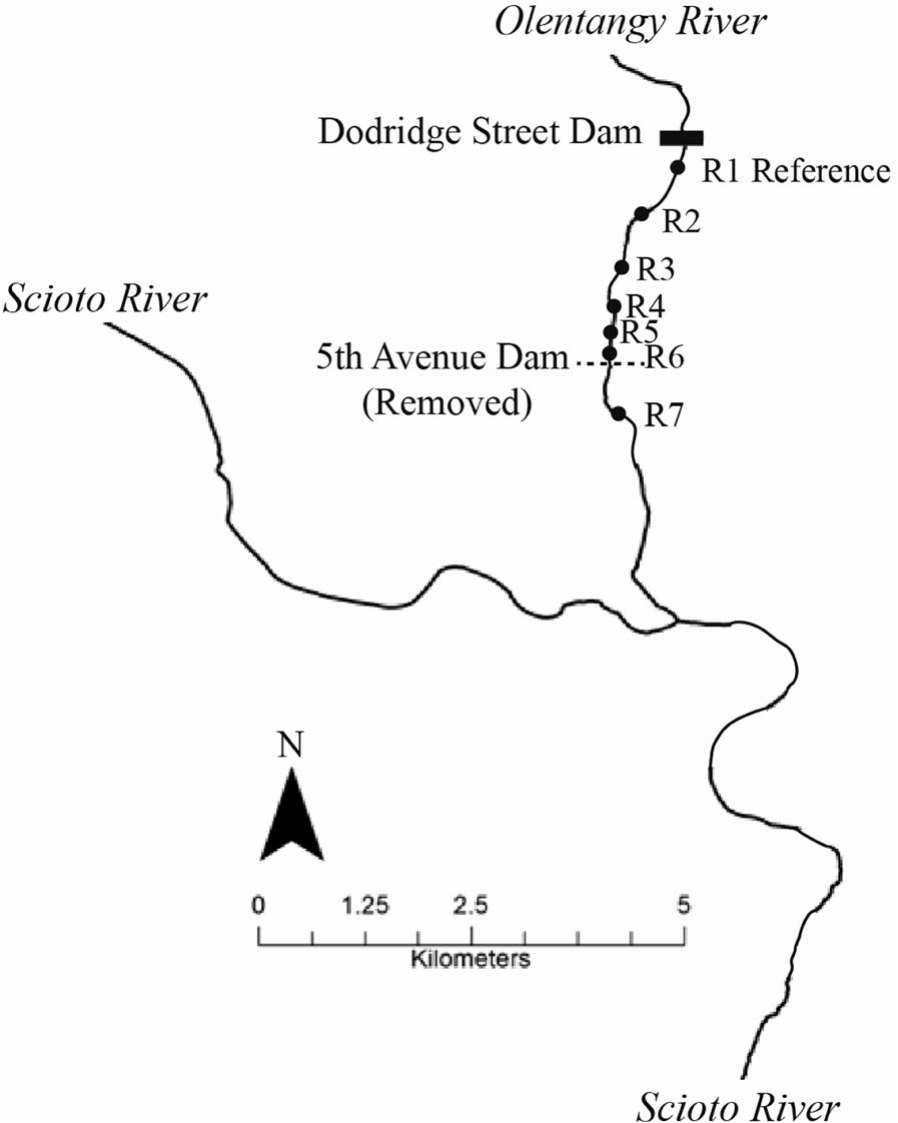
Sample riffles (filled circles) upstream and downstream of the previous 5th Avenue dam, Columbus, Ohio. Five riffles developed since dam removal (i.e., riffles 2 through 6). Riffle 1 was immediately downstream of an existing lowhead dam of similar size and age to the removed 5th Avenue dam and was sufficiently far upstream of the previous 5th Avenue dam to be considered non-impounded (free-flowing) (Stantec 2010). Thus, this riffle served as our reference reach. Riffles 2 and 3 were located in an actively restored section of the river following dam removal (i.e., natural channel design). Riffle 7 also existed before dam removal

The study area was a 3-river kilometer (rkm) section of the lower Olentangy River, a tributary of the Scioto River in central Ohio (Fig. 1). The Olentangy River is a mixed-bed river, comprised mostly of gravel and cobble. The 5th Avenue dam (143.3 m wide and 2.5 m high) was removed in late summer 2012 in order to improve water quality and aquatic habitat. Restoration efforts associated with the dam removal included channel restoration at sections of a 2.6-km segment upstream of the previous dam. Two of our study riffles (riffles 2 and 3, Fig. 1) were included in an actively restored section, where natural channel design was used to narrow channel width, plant riparian vegetation, and redevelop and reconnect floodplain wetlands allowing the river to regain the more natural form and functions that existed pre-dam construction (see Ohio EPA 2011 for additional details). Among other objectives, goals of channel restoration activities included achieving Warmwater Habitat designation for fish (as defined by the Ohio EPA), increasing both fish and macroinvertebrate community diversity, and meeting the classification of a C4 channel as described in Rosgen (1994: riffle-pool sequence with predominantly gravel substrate, gentle slope, and point bars with well-defined floodplain). Before dam removal, no riffles were present in the impounded sections of the river (i.e., the area aligning to location of riffles 2–6 post-dam removal; Fig. 1). Sediments in the impounded area upstream of previous dam were poorly sorted and consisted of sand (58%), ≥ gravel (35%), and silt/clay (7%) (Stantec 2010). Before dam removal, riffle 7 (downstream of the previous dam; Fig. 1) was well developed with predominantly gravel and cobble substrate.

In total, we surveyed five riffles upstream of the previous dam location, one riffle below the previous dam location, and one riffle downstream of an existing lowhead dam in the same river, which served as a reference site (Fig. 1). Within each riffle, three quadrats were established at the upstream, middle, and downstream portions of the riffle to characterize representative microhabitats based on flow and substrate characteristics. Fish, benthic macroinvertebrate, chemical water-quality, substrate, and streamflow surveys were conducted within each quadrat at six time intervals: late spring (June), summer (August), and late fall of 2014; early spring (March), late spring (June), and summer (August) of 2015.

This is not a before-after study; in fact, riffles were non-existent (or obscured) in the impounded area before dam removal, making comparable benthic macroinvertebrate collections not feasible at riffles 2–6. However, we collected macroinvertebrates downstream of the dam before removal (riffle 7). We also include benthic macroinvertebrate data from the Ohio Environmental Protection Agency from both before and after dam removal for both our reference site (Ohio EPA 1999, 2005; Mike Bolton, Ohio EPA personal communication) (riffle 1; Fig. 1). These “before” data were used as points of reference only and were not included in subsequent analyses.

### Physical habitat

For each quadrat at each sampling period, Wolman’s (1954) pebble-count method was used to estimate bed grain-size distribution with 200 haphazardly selected clasts measured using a gravelometer (e.g., D_50_ = particle size for which 50% of the particles are finer). Size categories (diameter) were as follows: fines (< 0.25 mm), sand (0.25–2.0 mm), gravel (2–16 mm), pebble (16–64 mm), cobble (64–256 mm), boulder (> 256 mm), and bedrock (solid surface). Relative grain roughness for each quadrat was calculated using the ratio of streamflow depth divided by D_84_ (i.e., 84th percentile of sediment distribution). Streamflow velocity (m s^−1^) was measured at two-thirds water depth using a FlowMate Model 2000 (Marsh-McBirney, Loveland, Colorado). Depth (m) was measured with a stadia rod as distance from the water surface to the substrate. Additionally, at the beginning and near the end of the study (late spring of 2014 and 2015), two cross-sectional profiles and one longitudinal survey of each riffle were conducted to determine mean channel slope (m m^−1^) using a total station (Gowin TKS-202, Beijing, China).

### Chemical water quality and nutrients

Temperature, conductivity, dissolved oxygen (DO), and pH were measured using a YSI 650 MDS® (YSI Inc., Yellow Springs, Ohio) with attached 600R® sonde at each quadrat during each sampling period. In addition, one 500-ml water sample was collected from each riffle (at middle of the thalweg) during each sampling period for total mercury (Hg), total nitrogen (N), total phosphorus (P), nitrate (NO_3_), phosphate (PO_4_), and ammonia nitrogen plus phosphate (NH_4_ + PO_4_). The samples were stored at 4 °C and sent for analysis at The Ohio State University Service, Testing, and Research (STAR) Laboratory (Wooster, Ohio), which follows standard methods and QA/QC protocols.

### Benthic macroinvertebrates and fish

At each quadrat and time interval, a 0.093 m^2^ (hereafter reported as 0.10 m^2^) Surber sampler (500-μm mesh size) was used to collect benthic macroinvertebrates (90 s per collection) from the stream bottom following Sullivan and Watzin (2008). Macroinvertebrates were stored in 70% ethanol and subsequently sorted from substrate material, identified to family using Merritt et al. (2008) as a guide, and enumerated.

Fish assemblages were surveyed within each quadrat at each time interval using a Smith-Root® LR-24 (Vancouver, Washington) backpack electrofisher under normal flow conditions. To prevent fish from leaving the quadrat, a frame with a weighted net (4.76-mm mesh) was deployed around the edge of the quadrat (modified from Bain et al. 1985). Pulling on upstream and downstream release cords enabled us to remotely set the frame net into final position. A time delay of 15 min between setting the frame and sampling the quadrat permitted a period without disturbance prior to sampling (Bain et al. 1985). One electrofishing pass of 100 s for each quadrat was conducted (total of 300 s per riffle). After collection and following enumeration and identification to species, fish were released.

### Numerical and statistical analysis

Family richness, evenness (*J’*), Simpson’s Index (1-*D*), and density were calculated for benthic macroinvertebrates. Due to low fish numbers, only species richness, density, and number of darter species were calculated for fish assemblages. These metrics were calculated for each quadrat as well as for each riffle for each time period. Species (or family) evenness (*J’*) quantifies the relative abundances of species/families within the assemblage and ranges from 0 to 1 where communities with an equitability number closer to 1 represent greater evenness (Pielou 1975). Simpson’s Index of Diversity (1-*D*) also ranges between 0 and 1; values closer to 1 indicate greater sample diversity (Simpson 1949; Pielou 1969). The index represents the probability that two individuals randomly selected from a sample will belong to different species, or in this case, family.

Given the spatial distribution of our study sites in the same river, we tested for potential spatial autocorrelation (Moran’s *I*) among response variables (macroinvertebrate and fish density, richness, and evenness) and found no evidence for non-random spatial patterns (*p* > 0.05 in all cases) (Moran 1950). All data were transformed (log [*x* + 1]) where necessary to meet assumptions of normality and homogeneity of variance. Linear mixed-effects models were used to test for differences in fish and benthic macroinvertebrate metrics among riffles over time, as well as for differences in streamflow velocity, mean water depth, and sediment-size distribution (D_16_ and D_50_) and roughness. Following Davis et al. (2017), time and riffles (i.e., “sites”) were included as fixed effects; quadrats were nested within study riffles and considered a random effect. For water-chemistry parameters, for which we only had one sample per reach per time period, one-way analysis of variance (ANOVA) was used to compare all reaches by time steps. Simple regression analysis was used to explore potential relationships between (1) roughness, D_50_, and changes in channel slope and benthic macroinvertebrate and fish metrics; (2) benthic macroinvertebrate density and fish density. Mixed models, regression analyses, and ANOVA were performed using JMP 11.0 (SAS Institute, Cary, North Carolina).

Non-metric multidimensional scaling (NMS) ordinations and analysis of similarity (ANOSIM; *α* = 0.05; 999 permutations) similar to Poulos et al. (2014) were used to examine differences in macroinvertebrate and fish community composition among sites and time steps. NMS enabled visualization of differences in assemblage structure among the riffles at the different times and was conducted separately for benthic macroinvertebrate and fish assemblages using 500 randomizations and Jaccardian distance matrices (scaled by variance to provide more equal weight to less abundant species/families), which are generally preferred for abundance data so that double absences do not contribute toward distance determination (Legendre and Legendre 1998). In NMS, distance matrices are rank-ordered and the solution determined iteratively by minimization of the stress criterion (Kruskal 1964). The ANOSIM statistic *R* denotes the magnitude of the difference among groups; *R* equals 1 when groups differ completely and equals 0 when there is no difference detected among groups.

Redundancy analysis (RDA) was used to identify potential differences in community composition of fish and benthic macroinvertebrate assemblages as a function of hydrogeomorphic and water-chemistry predictors among riffles (i.e., sites) and over time. To avoid the number of metrics exceeding the number of sites, the analysis was limited a priori to four metrics (dissolved oxygen [mg L^−1^], average streamflow velocity [m s^−1^], D_50_ [mm], and PO_4_ [mg L^−1^]). We used these metrics because of their importance to benthic macroinvertebrates and riffle fishes (Kessler and Thorp 1993; Paul and Meyer 2001) and because they reasonably represented the variability observed in the broader set of streamflow and water-chemistry parameters surveyed as part of this study. Thus, RDA is useful for distinguishing the effects of dam removal on macroinvertebrate and fish assemblages in terms of drivers related to either hydrogeomorphology (average stream flow velocity, D_50_) or chemical water quality (DO, PO_4_). We used R (R Statistical Computing Software, Vienna, Austria) for ordinations and ANOSIM (R Core Team 2016).

## Results

Sediment-size distribution, water depth, and streamflow velocity varied by riffle and over time. The density and diversity of macroinvertebrates and fish were also different over time, largely as a function of season. Macroinvertebrate assemblage composition was different by time but not riffle, whereas fish assemblages were similar irrespective of time or riffle. Both hydrogeomorphic characteristics and chemical water-quality parameters emerged as potential drivers of macroinvertebrate and fish assemblages.

### Physicochemical characteristics

Average values (± 1 SD) of water temperature, pH, and conductivity were 18.5 (± 6.6 °C), 8.40 (± 0.33), and 0.615 (± 0.148 μm cm^−1^), respectively, across the seven study riffles through all time steps (Supplementary Material: Table S1). Riffle 7, downstream of the previous dam, generally exhibited the highest water temperatures as compared to the other riffles over time. Total N and NO_3_ were lowest in August 2015 and November 2014 and highest in June 2015 (see Supplementary Material: Fig. S1). Total P and PO_4_ were more consistent over time, but still exhibited differences among time periods (note the high PO_4_ concentration at riffle 1 in August 2014).

**Fig. 2:**
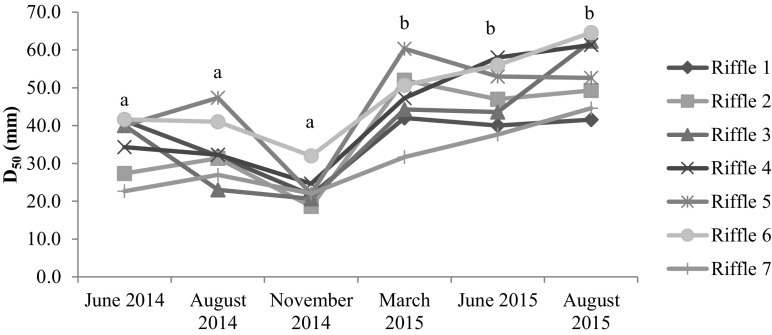
Median sediment size, D_50_, by study riffle over time (*F*_5,70_ = 12.44, *p* < 0.0001). Riffle 1 is the reference riffle, riffle 2–6 are upstream (of previous dam), and riffle 7 is downstream of the previous dam. Significant pairwise differences are indicated by different letters a and b (Tukey’s HSD: *p* < 0.05). For visual clarity, error bars are not included. However, please see Supplementary Material: Table S1 for details on data variability

**Fig. 3:**
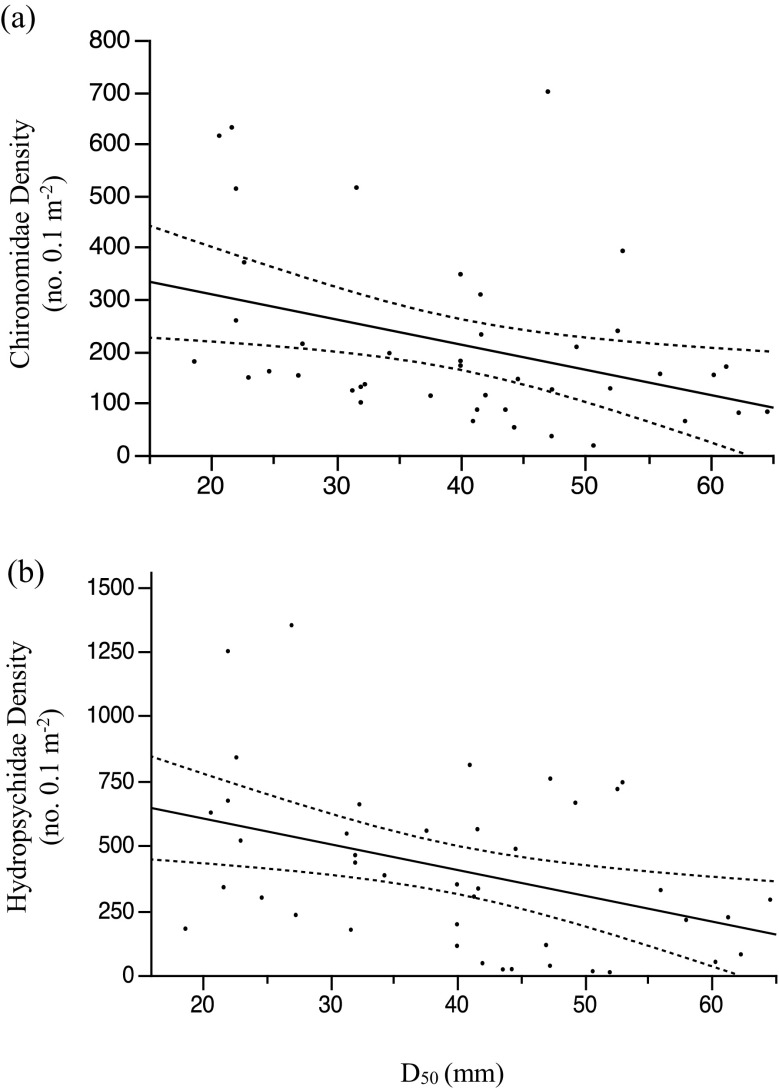
Relationships between D_50_ and densities (no. 0.1 m^−2^) of **a** Chironomidae (*y* = 406.8–4.87*x*; *R*^2^ = 0.14, *p* = 0.015) and **b** Hydropsychidae (*y* = 802.9–9.95*x*, *R*^2^ = 0.16, *p* = 0.008). Dashed lines represent confidence curves at *α* = 0.05

Across the study riffles and time periods, gravel ranged from 52.7 to 83.0%, and cobble ranged from 17.3 to 47.0%. Riffle 2 coarsened the most during the study period. The substrate composition of our reference site—riffle 1 (downstream riffle of an existing dam)—remained fairly consistent across the study period. D_16_ and D_50_ varied among study riffles and through time (Table 1, Fig. 2). D_16_ increased by 8.7 mm from June 2014 to June 2015 and by 12.8 mm from August 2014 to August 2015 (Supplementary Material: Table S1). D_50_ also increased from June and August 2014 to June and August 2015 (Fig. 2). D_50_ was negatively associated with densities of both Chironomidae (Fig. 3a) and Hydropsychidae (Fig. 3b).

**Table 1:** Linear mixed-effects models for fish and benthic macroinvertebrate response variables. “Site” = study riffle. Also included are D_16_ and D_50_, relative roughness, streamflow velocity, and mean water depth

Source	*df*	*F*	*p*
Benthic macroinvertebrates			
Density (no. 0.1 m^−2^)			
Site	6, 14	6.24	0.002
Time	5, 70	12.64	< 0.0001
Site*time	30, 70	1.50	0.084
Simpson’s *(1-D)*			
Site	6, 14	1.50	0.249
Time	5, 70	3.36	0.009
Site*time	30, 70	0.82	0.722
Family richness			
Site	6, 14	2.16	0.111
Time	5, 70	19.45	< 0.0001
Site*time	30, 70	3.97	< 0.0001
Evenness (*J*’)			
Site	6, 14	1.93	0.146
Time	5, 70	7.45	< 0.0001
Site*time	30, 70	0.79	0.753
Fish			
Density (no. 2.25 m^−2^)			
Site	6, 14	1.58	0.225
Time	5, 70	3.47	0.007
Site*time	30, 70	0.51	0.976
Species richness (*S*)			
Site	6, 14	1.24	0.345
Time	5, 70	3.80	0.004
Site*time	30, 70	0.52	0.974
Darter species richness			
Site	6, 14	0.93	0.501
Time	5, 70	3.89	0.004
Site*time	30, 70	0.74	0.820
Hydrogeomorphology			
D_16_ (mm)			
Site	6, 14	3.05	0.040
Time	5, 70	31.67	< 0.0001
Site*time	30, 70	2.05	0.007
D_50_ (mm)			
Site	6, 14	7.08	0.001
Time	5, 70	12.44	< 0.0001
Site*time	30, 70	0.76	0.791
Relative roughness			
Site	6, 14	2.64	0.063
Time	4, 56	3.51	0.013
Site*time	24, 56	1.18	0.295
Streamflow velocity (m s^−1^)			
Site	6, 14	1.50	0.219
Site*time	24, 56	1.31	0.203
Average depth (m)			
Site	6, 14	1.40	0.251
Time	4, 56	9.08	< 0.0001
Site*time	24, 56	0.76	0.756

Streamflow velocity ranged from 0.04 to 1.50 m s^−1^ ($\overline{x}$ = 0.50) across all riffles through time and was significantly different over time (Table 1), which was expected because of seasonal differences in hydrology (Supplementary Material: Table S1). The lowest flows occurred in March 2015, and the highest occurred in June 2015 (although note missing values from November 2014). Water depth ranged from 0.04 to 0.85 m ($\overline{x}$ = 0.19 m) across all riffles and time periods and also varied significantly across time (Table 1, Supplementary Material: Table S1).

Mean channel slope was 0.004 m m^−1^ across all riffles in June 2014 and 0.008 m m^−1^ in June 2015. The change in channel slope from 2014 to 2015 (Δ slope) was positively related to benthic macroinvertebrate family richness (Supplementary Material: Fig. S2a) but negatively related to fish species richness (Supplementary Material: Fig. S2b) and density (Supplementary Material: Fig. S2c). Relative bed roughness was significantly different through time (Table 1), with the greatest decrease in roughness at riffle 4 and the largest increase in roughness at riffle 7. We observed no relationship between the change in roughness and benthic macroinvertebrate or fish density or diversity (*p* > 0.05, data not shown).

### Biotic assemblages

**Fig. 4:**
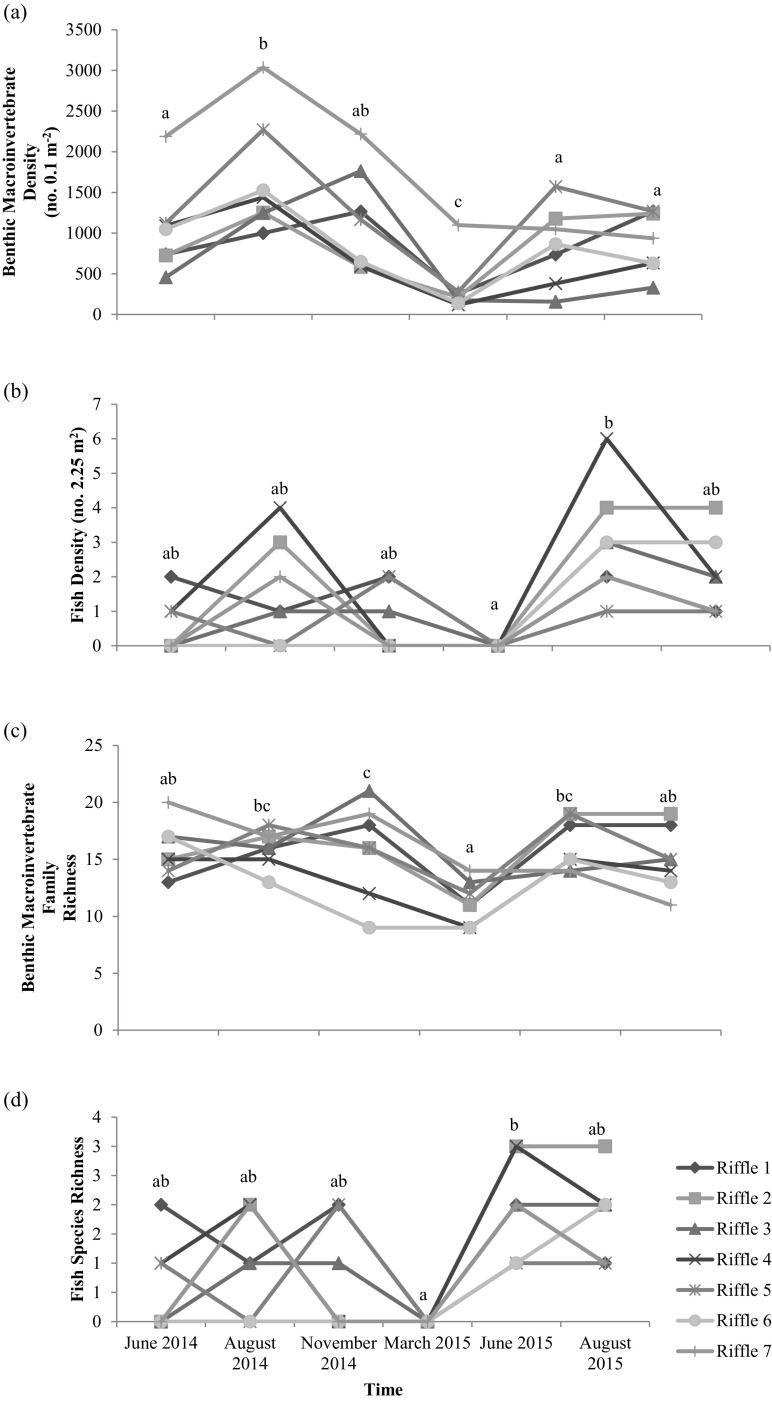
Benthic macroinvertebrate and fish assemblage responses by study riffle over time. **a** Benthic macroinvertebrate mean density (no. 0.1 m^−2^) (*F*_5,70_ = 12.64, *p* < 0.0001). **b** Fish density (no. 2.25 m^−2^) (*F*_5,70_ = 3.47, *p* = 0.007). **c** Benthic macroinvertebrate family richness (*F*_5,70_ = 19.45, *p* < 0.0001). **d** Fish species richness (*F*_5,70_ = 0.52, *p* = 0.975). Riffle 1 is the reference riffle, riffles 2–6 are upstream (of previous dam), and riffle 7 is downstream of the previous dam. Significant pairwise differences based are indicated by different letters a, b, and c (Tukey’s HSD: *p* < 0.05). For visual clarity, error bars are not included. However, please see Supplementary Material: Table S1 and Fig. S3 for details on data variability

Benthic macroinvertebrate density averaged 332.6 individuals 0.1 m^−2^ across all riffles through time. Macroinvertebrate density was significantly different among riffles (Table 1), with riffle 7 exhibiting the greatest density ($\overline{x}$ = 1754.2 individuals 0.1 m^−2^; Supplementary Material: Fig. S3). For comparison, density was 2544 individuals 0.1 m^−2^ just prior to dam removal in August 2012. Macroinvertebrate density was also different over time (Table 1; Fig. 4a). As a point of reference, macroinvertebrate abundance at riffle 1 (as assessed by the Ohio EPA) was variable between 1987 and 2011, and the abundance in 2015 was within the previous range and on par with 1999 and 2004 (Supplementary Material: Fig. S4).

Fish density across all riffles at all time steps averaged 0.4 individuals 2.25 m^−2^ (Supplementary Material: Table S1). Fish density was also significantly different by time (Fig. 4b). Fish density was not different among study riffles (Table 1). There was no relationship between benthic macroinvertebrate density and fish density at any of the study riffles or time steps (*p* > 0.05, data not shown).

Benthic macroinvertebrate family richness ranged from 3 to 17 ($\overline{x}$ = 10.8) across all riffles through time (Supplementary Material: Table S1). Thirty insect families were represented in 12 orders, as well as class Oligochaeta and phylum Platyhelminthes. The most abundant families were Hydropsychidae, Chironomidae, and Baetidae, comprising 41, 21, and 11% of the total number of individuals collected over the study, respectively. Benthic macroinvertebrate richness was lowest in March across the year, but was comparable across the study riffles in June 2014 and June 2015 (Fig. 4c). Across six of the seven sites (except riffle 1), there was a decrease in macroinvertebrate density from August 2014 to August 2015. Macroinvertebrate richness was not different among study riffles, but was significantly different through time (Table 1). Linear mixed models also indicated a significant interaction effect between time and study riffle (*p* < 0.0001) for macroinvertebrate richness, with the effect of time greater for riffles 1, 2, and 5 than for the others. Macroinvertebrate evenness also varied significantly over time (Supplementary Material: Table S1), showing greatest evenness at riffle 4 and lowest at riffle 6 (data not shown). Simpson’s Index for macroinvertebrates was not significantly different among study riffles over time (Table 1).

Fish species richness and darter species richness both ranged from 0 to 3 by study riffle. Richness of all fish species (Fig. 4d) and darter species (*p* = 0.004; data not shown) varied temporally across the riffles, but not among study riffles (Table 1). The most common darter species included Banded (*Etheostoma zonale*) and Rainbow Darters (*E. caeruleum*), occurring in five and six of the study riffles, respectively. Rainbow and Banded Darters were found at all study sites except for riffle 7. Greenside Darters (*E. blennioides*) were only found at riffle 1. Bluebreast Darters (*E. camurum*) were only observed in August 2015 at riffles 2 and 4. Neither Simpson’s Index nor evenness of fish assemblages varied among riffles or over time (Table 1).

### Shifts in assemblage structure

**Fig. 5:**
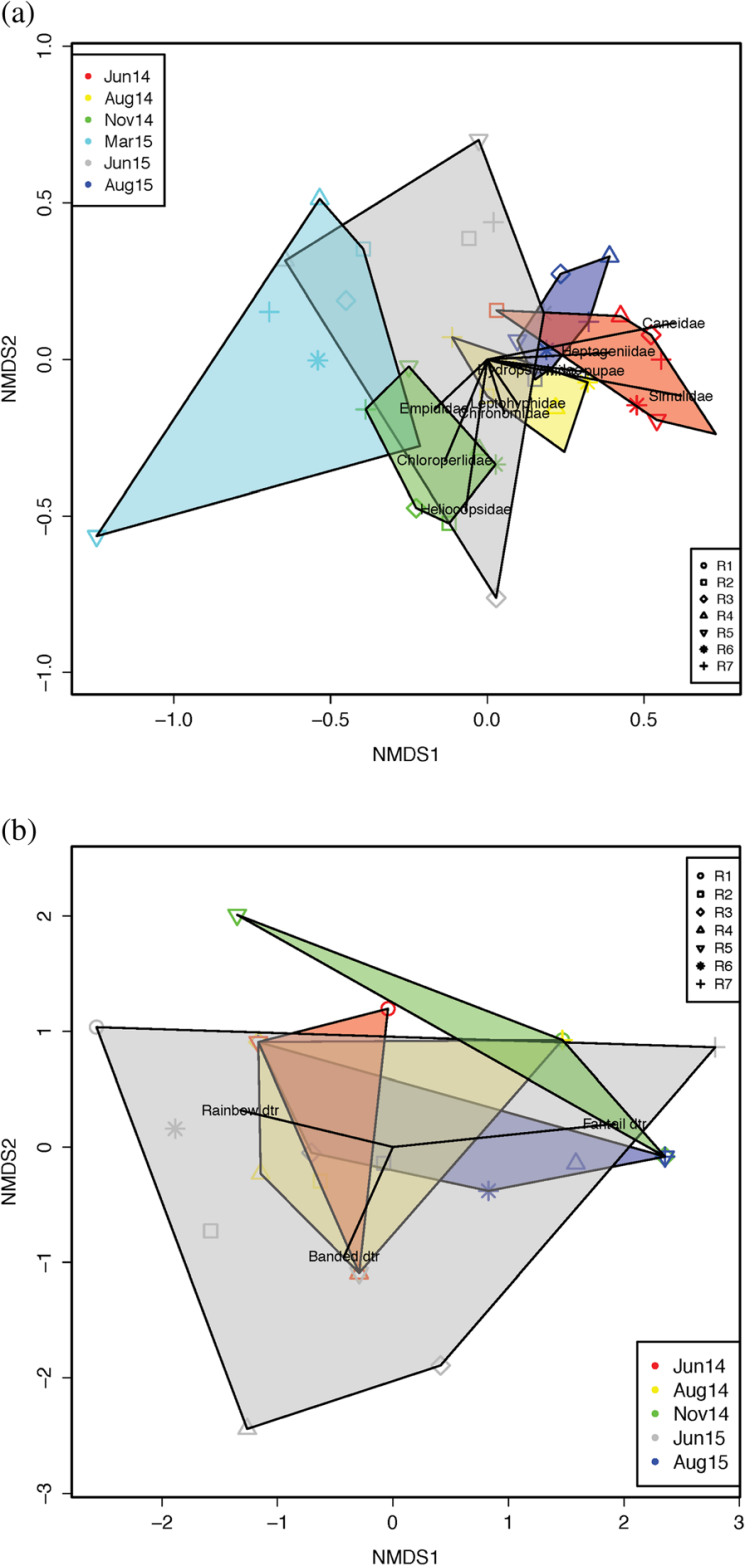
Non-metric multidimensional scaling (NMS) ordination plots of **a** benthic macroinvertebrate and **b** fish assemblage compositions grouped by date (scaled by variance). The stress values were 0.22 and 0.08, respectively. The different shapes indicate the different riffles and the different colors indicate the different sampling time periods; only the most significant families are indicated. Dates: June 2014 = red, August 2014 = yellow, November 2014 = green, March 2015 = cyan, June 2015 = gray, August 2015 = blue. Note that there are differences in the temporal representation of fish data in **b** (i.e., no fish were found in March 2015 and August 2015); otherwise, colors of the polygons are as noted in **a**. Riffles are shown by symbol: riffle 1 = circle, riffle 2 = square, riffle 3 = diamond, riffle 4 = triangle (up), riffle 5 = triangle (down), riffle 6 = asterisk, riffle 7 = plus. Dtr. darter

NMS ordination separated benthic invertebrate assemblage composition by time but not by study riffle along NMS1 (axis 1), which was confirmed by analysis of similarity (ANOSIM—time: *R* = 0.588, *p* = 0.001; site/riffle: *R* = 0.012, *p* = 0.371) (Fig. 5a). For fish assemblages, there was no difference in composition by time (Fig. 5b), or by site (time: *R* = 0.026, *p* = 0.347; site/riffle: *R = −* 0.028, *p =* 0.570).

Across all study riffles, there was a large proportion of unconstrained variance identified by RDA. For species and site scores, species were scaled proportionally to associated eigenvalues, while sites remained unscaled. Across all study riffles over time, RDA showed that benthic macroinvertebrate abundance had a weak positive association with D_50_ (data not shown). PO_4_ was at least weakly (and positively) related to benthic macroinvertebrate density in all but riffles 1 and 5 (mostly in late spring and summer; see Supplementary Material: Fig. S5). DO positively aligned with benthic macroinvertebrate family richness (riffles 1, 2, and 6; see Supplementary Material: Fig. S5a, b, f) but not density (but note negative relationship at riffle 5; Supplementary Material: Fig. S5e) for certain time periods. Of the physicochemical variables, streamflow velocity emerged as the most influential physicochemical variable from the RDA, where it aligned with macroinvertebrate family richness or density at multiple riffles, but was inconsistent relative to the nature of the association (Supplementary Material: Fig. S5).

**Fig. 6:**
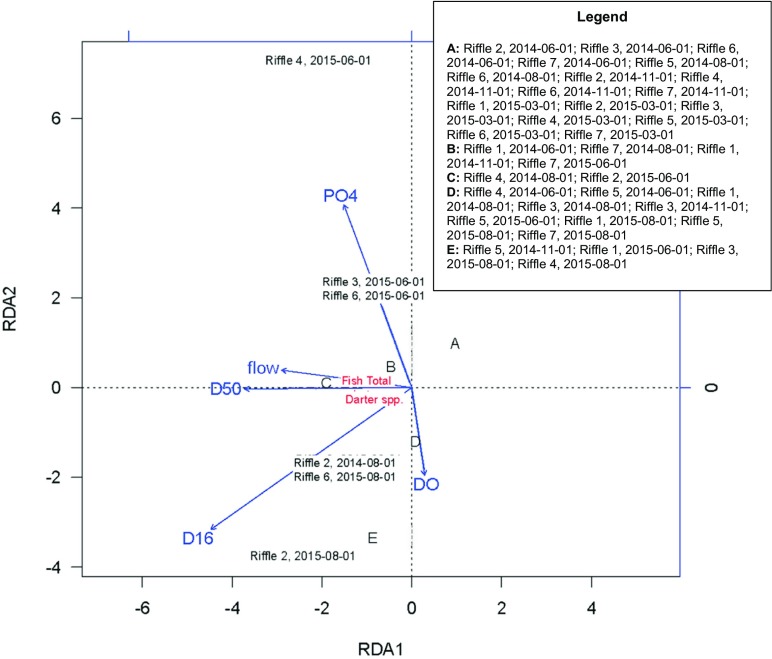
Redundancy analysis (RDA) of fish density (fish total), and darter species richness (Darter spp) at each study riffle across all the time steps. Blue arrows indicate how environmental variables were ordinated. Overall, there were 42 riffle/date combinations but only 9 unique scores. Where possible, riffles and time steps (e.g., Riffle 3, 2015-06-01) are included in the plot. For locations where labels would overlap and be illegible, letters A, B, C, D, and E represent the respective riffles and time steps as listed in the legend

Although we detected no differences in fish assemblages by time or riffle, fish and darter species richness were positively associated with D_16_, D_50_, and streamflow velocity at multiple riffles and time periods (e.g., riffles 1, 2, 3, 5, 7); fish metrics were negatively (but weakly) related to DO (Fig. 6).

## Discussion

### Riffle development following dam removal

Altered streamflow patterns characteristic of impounded rivers destroy riffles within the reservoir pool and impede the maintenance of riffles both further upstream as well as downstream. After dam removal, new riffles can form in previously impounded reaches, although these bedforms may exhibit low habitat diversity compared to reference riffles (Burroughs et al. 2009). We observed that five riffles had developed upstream of the previous dam (in the former pool and its tail; riffles 2–6). Two of the upstream reaches were within an area of active channel restoration, likely leading to rapid development. Similar to other studies (e.g., following removal of ~ 2 m high Manatawny Creek Dam, Pennsylvania; Bushaw-Newton et al. 2002), we also documented a coarsening of bed sediment: average gravel composition decreased through time from 70.4 to 60.3% across all riffles, while average cobble composition increased from 29.4 to 39%. We observed no shift in grain-size distribution at the reference site (riffle 1).

Increases in average substrate size in formerly impounded areas are generally in response to higher slope and greater streamflow velocities (Burroughs et al. 2009). In addition to increases in D_50_, slope also increased from June 2014 to June 2015 across the riffles (although note there was substantial variability among sites). Similar to findings by Cooper (2013), variability in streamflow velocity was also likely a key driver of riffle development in our system, and the interactions among these variables are potentially critical.

The six upstream riffles (riffles 1–6) exhibited greater median particle size (D_50_) than the downstream riffle (riffle 7) (but note that riffle 7 increased through time as well). This finding is similar to that reported by Thomson et al. (2005), who found that particle size was reduced in the downstream riffles after the Manatawny Creek Dam removal. Conversely, Kanehl et al. (1997) reported that mean percentage rocky substrate increased in the formerly impounded areas through time (~ 4 years) following removal of a lowhead dam on the Milwaukee River, yet sediment size (reported as the average across the study site) remained similar over time downstream of the previous dam site. Thus, smaller particle size in our downstream reach (riffle 7) supports our predictions and suggests at least some degree of downstream transport of fine sediment from the previous impoundment, despite evidence that the transport of sediment over lowhead dams is not fully restricted owing to their small size (e.g., Stanley et al. 2002; Tullos et al. 2014).

### Benthic macroinvertebrate and fish assemblages

Our results were similar to those of Poulos et al. (2014), who documented changes in biotic communities in both riffles upstream and downstream of the previous lowhead dam. As anticipated, we observed strong seasonal changes in macroinvertebrate density and richness (Fig. 4a, c). Macroinvertebrate density, but not richness or assemblage structure, varied by study riffle with riffle 7 generally exhibiting the highest densities through time. However, density at this site still was lower than just before dam removal and potentially linked to an overall fining of the sediment in this reach following dam removal. Fine sediments commonly increase embeddedness (percent of fine sediment surrounding large gravel and cobbles), which can reduce benthic-insect abundances (Nerbonne and Vondracek 2001) and is a likely mechanism for the lower macroinvertebrate density at riffle 7 following dam removal. For comparison, the dominant taxa in impounded areas before dam removal were midges (Chironomidae) and aquatic worms, whereas following dam removal Hydropsychidae, Chironomidae, and Baetidae represented the core macroinvertebrate community (Stantec 2010 and references therein). Maloney et al. (2008) also report a shift from impounded to free-flowing macroinvertebrate assemblages following removal of the South Batavia dam (105 m wide, 2.7 m high) on the Fox River, Illinois.

Overall, we found limited evidence to support our hypotheses that hydrogeomorphic characteristics would relate to macroinvertebrate density and diversity, despite substantial evidence for links between stream hydrogeomorphic features and macroinvertebrates (Sullivan et al. 2004; Bey and Sullivan 2015; Friberg et al. 2009). Across the study reaches, D_50_ was negatively related to specific families of insects (e.g., Chironomidae), which is consistent with known tolerance of Chironomidae assemblages to sediment pollution (Zweig and Rabeni 2001; Carew et al. 2007, but note that specific chironomid taxa may be more sensitive to shifts in sediment). We also observed associations between macroinvertebrates and both substrate size and streamflow velocity (from the RDA), but the variable nature of the relationships (i.e., a mixture of positive and negative associations with macroinvertebrate density or family richness) makes interpretation difficult. Associations between water chemistry/nutrients and macroinvertebrate communities were strongest for PO_4_ (and limited evidence for DO; Supplementary Material: Fig. S5). Within the concentration ranges observed in this study, positive relationships observed between PO_4_ and macroinvertebrate density may be a result of increased grazing opportunities without leading to toxicity or eutrophication levels. Additional associations might have been expected if the range of water-chemistry values had approached toxicity thresholds.

Fish density increased significantly from June 2014 to June 2015 in our study, even though overall numbers were relatively low compared to riffles in other systems in the area (e.g., 1.5 individuals m^−2^ in Big Darby Creek, Ohio [Bey and Sullivan 2015] vs. 0.5 individuals m^−2^ in this study). Bushaw-Newton et al. (2002) found an increase in riffle fish species downstream of a former dam, but we observed no difference in species richness across study riffles. Multiple darter species colonized the newly developed riffles (as well as a few additional benthic insectivores, e.g., Johnny Darter [*Etheostoma nigrum*] at riffle 4), although darter richness was consistently greatest at our reference site. Schwartz and Herricks (2007) found that riffle fishes (i.e., darters and some cyprinids) remained absent after construction of pool-riffle structure in an urban Illinois stream, implicating lack of macroinvertebrate food sources (rather than habitat) as the limiting factor for fish colonization. Although macroinvertebrate density was related to substrate size (Fig. 3) and varied seasonally (Fig. 4c), there was no difference among upstream study riffles and no relationship between macroinvertebrate and fish densities. Thus, in our study, food resource limitation was an unlikely driver of variability in riffle fish abundance.

Fish density and darter species richness were related to streamflow velocity (which was relatively consistent across time, although there was variability among study riffles; Supplementary Material: Table S1) and D_16_ and D_50_ in our RDA for multiple sites, but did not align strongly with water-chemistry parameters (Fig. 6). This is in contrast to Hering et al. (2006), who found that fish responded more strongly to nutrient enrichment then to land use, hydrogeomorphology (reach and microhabitat scales), or a habitat-degradation gradient in a comparison of 185 streams across Europe. In addition to the direct effects of substrate and variability in streamflow velocity on both macroinvertebrates and fishes (Greenberg 1991; Heino 2004; McQuist and Schultz 2014), hydrogeomorphic processes might be expected to exert indirect influences on biota via by controlling the dynamics of dissolved N and P (Velinsky et al. 2006) and suspended sediment (Kemp et al. 2011).

Although we recorded snapshots of variability in streamflow velocity and water depth over time, our study is constrained by the lack of continuous measures of these variables, and this will be an area of important research relative to riffle-biota associations following dam removal. Tracking and assessing responses to high-flow disturbances may also further illuminate mechanistic drivers. For example, both macroinvertebrates and fish appeared to respond to a scouring event in March 2015 with recovery by June 2015 (Fig. 4), providing initial evidence that more focused research on the impacts of critical flow events on linked geomorphic-biotic responses following dam removal may be warranted.

### Conclusions

We observed differences in both benthic macroinvertebrate and fish assemblages among riffles and over the 15 months of this study, with the strongest gains in diversity and density in riffle fish assemblages. Our findings suggest that riffle formation and the associated biotic responses following dam removal are complex and require consideration of both chemical and physical water-quality characteristics. They also provide initial evidence of the benefits of riffle habitat structures as part of dam removal restoration efforts in gravel-bed rivers, supporting the importance of riffle morphology for aquatic biodiversity (Brooks et al. 2005; Costa and Melo 2008; Cianfrani et al. 2009).

Few studies have focused on fine-scale effects of dam removal (e.g., riffle habitat units) on linked physical-biotic responses, yet this level of resolution may be an important step in further understanding ecosystem responses to lowhead dam removal. Interdisciplinary and longer term (> 5 years) monitoring of ecological responses to dam removals in varying habitats and stream types will provide a more holistic understanding of post-dam removal ecosystem changes. Additionally, further evaluation of fish and benthic macroinvertebrate habitat responses to dam removal will be necessary in order to further inform fish conservation strategies as they relate to dam removal.

## Electronic supplementary material

ESM 1 (DOCX 1717 kb)
