# Supplementary material for: Associations between riffle development and aquatic biota following lowhead dam removal
Source: Environ Monit Assess. 2018 May 10;190(6):339. doi: 10.1007/s10661-018-6716-1 (PMC5945803; doi:10.1007/s10661-018-6716-1)
Supplement: Supplementary file 1 — (DOCX 1717 kb) [file 10661_2018_6716_MOESM1_ESM.docx]

**Supplementary Material**

**Journal:** Environmental Monitoring and Assessment

**Title:** Associations between riffle development and aquatic biota following lowhead dam removal

**Authors:** Danielle R. Cook^1^ & S. Mažeika P. Sullivan^1,2^

^1^Schiermeier Olentangy River Wetland Research Park, School of Environment and Natural Resources, The Ohio State University, Columbus, Ohio 43202

^2^Corresponding author: email – [sullivan.191@osu.edu](mailto:sullivan.191@osu.edu), telephone – (614) 688-8402, fax – (614) 292-7314. ORCID id: orcid.org/0000-0003-2341-5316

**Table S1.** Summary statistics of biotic metrics, hydrogeomorphic variables, and water-chemistry parameters through time for Olentangy River study riffles. Dashes indicate missing data.

|  |  | **Min** | | **Median** | | **Max** | **Mean** | | **SD** |
| --- | --- | --- | --- | --- | --- | --- | --- | --- | --- |
| **Biotic variables** | **Date** |  |  | |  | |  | |  |
| *Benthic Macroinvertebrates* |  |  |  | |  | |  |  | |
| *Density (no. 0.1 m^-2^)* | June 2014 | 74.0 | 303.0 | | 1,114.0 | | 351.0 | 247.1 | |
|  | August 2014 | 115.0 | 468.0 | | 1,363.0 | | 560.8 | 306.9 | |
|  | November 2014 | 59.0 | 330.0 | | 1,196.0 | | 392.5 | 292.1 | |
|  | March 2015 | 18.0 | 72.0 | | 644.0 | | 108.6 | 140.6 | |
|  | June 2015 | 3.0 | 281.0 | | 792.0 | | 282.6 | 213.6 | |
|  | August 2015 | 45.0 | 264.0 | | 616.0 | | 299.9 | 164.3 | |
|  |  |  |  | |  | |  |  | |
| *Simpsons (1-D)* | June 2014 | 0.552 | 0.745 | | 0.836 | | 0.748 | 0.063 | |
|  | August 2014 | 0.462 | 0.705 | | 0.849 | | 0.690 | 0.087 | |
|  | November 2014 | 0.138 | 0.647 | | 0.808 | | 0.620 | 0.157 | |
|  | March 2015 | 0.280 | 0.734 | | 0.826 | | 0.706 | 0.122 | |
|  | June 2015 | 0.472 | 0.669 | | 1.000 | | 0.684 | 0.114 | |
|  | August 2015 | 0.544 | 0.696 | | 0.833 | | 0.718 | 0.080 | |
|  |  |  |  | |  | |  |  | |
| *Family Richness* | June 2014 | 8.0 | 11.0 | | 15.0 | | 11.2 | 2.1 | |
|  | August 2014 | 9.0 | 12.0 | | 15.0 | | 12.0 | 1.6 | |
|  | November 2014 | 6.0 | 12.0 | | 14.0 | | 11.2 | 2.7 | |
|  | March 2015 | 4.0 | 8.0 | | 12.0 | | 7.8 | 2.0 | |
|  | June 2015 | 3.0 | 12.0 | | 17.0 | | 11.4 | 3.3 | |
|  | August 2015 | 8.0 | 11.0 | | 16.0 | | 11.3 | 2.1 | |
|  |  |  |  | |  | |  |  | |
| *Evenness (J’)* | June 2014 | 0.560 | 0.713 | | 0.824 | | 0.708 | 0.073 | |
|  | August 2014 | 0.454 | 0.604 | | 0.857 | | 0.609 | 0.099 | |
|  | November 2014 | 0.195 | 0.578 | | 0.766 | | 0.558 | 0.135 | |
|  | March 2015 | 0.332 | 0.747 | | 0.939 | | 0.736 | 0.140 | |
|  | June 2015 | 0.441 | 0.613 | | 1.000 | | 0.628 | 0.122 | |
|  | August 2015 | 0.506 | 0.688 | | 0.906 | | 0.688 | 0.101 | |
| *Fish* |  |  |  | |  | |  |  | |
| *Density (no. 2.25m^-2^)* | June 2014 | 0.0 | 0.0 | | 2.0 | | 0.2 | 0.5 | |
|  | August 2014 | 0.0 | 0.0 | | 3.0 | | 0.5 | 0.8 | |
|  | November 2014 | 0.0 | 0.0 | | 1.0 | | 0.2 | 0.4 | |
|  | March 2015 | 0.0 | 0.0 | | 0.0 | | 0.0 | 0.0 | |
|  | June 2015 | 0.0 | 1.0 | | 6.0 | | 1.0 | 1.4 | |
|  | August 2015 | 0.0 | 1.0 | | 2.0 | | 0.7 | 0.8 | |
|  |  |  |  | |  | |  |  | |
| *Species Richness (S)* | June 2014 | 0.0 | 0.0 | | 2.0 | | 0.2 | 0.5 | |
|  | August 2014 | 0.0 | 0.0 | | 2.0 | | 0.4 | 0.6 | |
|  | November 2014 | 0.0 | 0.0 | | 1.0 | | 0.2 | 0.4 | |
|  | March 2015 | 0.0 | 0.0 | | 0.0 | | 0.0 | 0.0 | |
|  | June 2015 | 0.0 | 1.0 | | 3.0 | | 0.8 | 0.9 | |
|  | August 2015 | 0.0 | 0.0 | | 2.0 | | 0.6 | 0.7 | |
|  |  |  |  | |  | |  |  | |
| *Darter Species Richness* | June 2014 | 0.0 | 0.0 | | 1.0 | | 0.1 | 0.4 | |
|  | August 2014 | 0.0 | 0.0 | | 3.0 | | 0.5 | 0.8 | |
|  | November 2014 | 0.0 | 0.0 | | 1.0 | | 0.2 | 0.4 | |
|  | March 2015 | 0.0 | 0.0 | | 0.0 | | 0.0 | 0.0 | |
|  | June 2015 | 0.0 | 1.0 | | 2.0 | | 0.6 | 0.6 | |
|  | August 2015 | 0.0 | 0.0 | | 2.0 | | 0.6 | 0.7 | |
|  |  |  |  | |  | |  |  | |
| **Hydrogeomorphology** |  |  |  | |  | |  |  | |
| *Streamflow Velocity (m s^-1^)* | June 2014 | 0.20 | 0.47 | | 1.18 | | 0.47 | 0.22 | |
|  | August 2014 | 0.09 | 0.47 | | 0.94 | | 0.45 | 0.19 | |
|  | November 2014 | −−− | −−− | | −−− | | −−− | −−− | |
|  | March 2015 | 0.04 | 0.44 | | 0.71 | | 0.43 | 0.19 | |
|  | June 2015 | 0.16 | 0.66 | | 1.50 | | 0.69 | 0.32 | |
|  | August 2015 | 0.09 | 0.49 | | 1.11 | | 0.44 | 0.28 | |
|  |  |  |  | |  | |  |  | |
| *Average Water Depth (m)* | June 2014 | −−− | −−− | | −−− | | −−− | −−− | |
|  | August 2014 | 0.04 | 0.16 | | 2.87 | | 0.37 | 0.71 | |
|  | November 2014 | 0.06 | 0.15 | | 0.24 | | 0.15 | 0.05 | |
|  | March 2015 | 0.08 | 0.20 | | 0.49 | | 0.20 | 0.09 | |
|  | June 2015 | 0.12 | 0.30 | | 0.85 | | 0.31 | 0.16 | |
|  | August 2015 | 0.05 | 0.15 | | 0.31 | | 0.17 | 0.08 | |
|  |  |  |  | |  | |  |  | |
| *D_16_ (mm)* | June 2014 | 6.4 | 9.4 | | 12.0 | | 9.7 | 1.5 | |
|  | August 2014 | 6.9 | 9.4 | | 14.0 | | 9.6 | 1.8 | |
|  | November 2014 | 6.3 | 8.5 | | 10.0 | | 8.4 | 1.1 | |
|  | March 2015 | 10.0 | 17.0 | | 33.0 | | 18.7 | 7.4 | |
|  | June 2015 | 4.4 | 18.0 | | 29.0 | | 18.4 | 6.1 | |
|  | August 2015 | 6.9 | 22.0 | | 49.0 | | 22.4 | 8.5 | |
|  |  |  |  | |  | |  |  | |
| *D_50_ (mm)* | June 2014 | 16.0 | 35.0 | | 50.0 | | 34.9 | 9.5 | |
|  | August 2014 | 16.0 | 32.0 | | 51.0 | | 33.4 | 9.6 | |
|  | November 2014 | 17.0 | 22.0 | | 33.0 | | 23.1 | 5.2 | |
|  | March 2015 | 26.0 | 45.0 | | 71.0 | | 46.9 | 14.2 | |
|  | June 2015 | 32.0 | 47.0 | | 61.0 | | 47.9 | 9.3 | |
|  | August 2015 | 30.0 | 49.0 | | 120.0 | | 53.8 | 19.2 | |
|  |  |  |  | |  | |  |  | |
| *Relative Roughness* | June 2014 | −−− | −−− | | −−− | | −−− | −−− | |
|  | August 2014 | 0.000 | 0.002 | | 0.029 | | 0.004 | 0.007 | |
|  | November 2014 | 0.001 | 0.002 | | 0.004 | | 0.002 | 0.001 | |
|  | March 2015 | 0.001 | 0.002 | | 0.005 | | 0.002 | 0.001 | |
|  | June 2015 | 0.001 | 0.003 | | 0.008 | | 0.003 | 0.002 | |
|  | August 2015 | 0.000 | 0.004 | | 0.014 | | 0.004 | 0.003 | |
| **Water Chemistry** |  |  |  | |  | |  |  | |
| *Temperature (°C)* | June 2014 | 21.08 | 23.57 | | 24.61 | | 23.08 | 1.21 | |
|  | August 2014 | 21.46 | 23.06 | | 25.40 | | 23.50 | 1.36 | |
|  | November 2014 | 8.28 | 9.08 | | 10.72 | | 9.15 | 0.73 | |
|  | March 2015 | 8.32 | 8.94 | | 12.04 | | 9.69 | 1.38 | |
|  | June 2015 | 19.98 | 21.78 | | 23.07 | | 21.64 | 0.83 | |
|  | August 2015 | 21.68 | 24.43 | | 26.17 | | 23.89 | 1.69 | |
|  |  |  |  | |  | |  |  | |
| *Conductivity ([mS cm^2^]^-1^)* | June 2014 | 0.467 | 0.637 | | 0.696 | | 0.611 | 0.072 | |
|  | August 2014 | 0.412 | 0.515 | | 0.577 | | 0.484 | 0.059 | |
|  | November 2014 | 0.017 | 0.685 | | 0.705 | | 0.615 | 0.169 | |
|  | March 2015 | 0.454 | 0.874 | | 0.908 | | 0.819 | 0.139 | |
|  | June 2015 | 0.521 | 0.527 | | 0.548 | | 0.529 | 0.007 | |
|  | August 2015 | 0.293 | 0.647 | | 0.708 | | 0.629 | 0.099 | |
|  |  |  |  | |  | |  |  | |
| *DO (mg L^-1^)* | June 2014 | 8.44 | 9.69 | | 13.16 | | 10.22 | 1.53 | |
|  | August 2014 | 7.07 | 9.06 | | 10.91 | | 8.93 | 1.18 | |
|  | November 2014 | 4.94 | 10.35 | | 15.24 | | 10.02 | 3.60 | |
|  | March 2015 | 0.17 | 11.43 | | 23.62 | | 10.83 | 7.62 | |
|  | June 2015 | 8.78 | 10.32 | | 11.31 | | 10.16 | 0.58 | |
|  | August 2015 | 9.04 | 12.13 | | 17.39 | | 12.29 | 2.65 | |
|  |  |  |  | |  | |  |  | |
| *pH* | June 2014 | 8.40 | 8.54 | | 8.86 | | 8.58 | 0.13 | |
|  | August 2014 | 7.74 | 8.03 | | 8.40 | | 8.02 | 0.14 | |
|  | November 2014 | 7.93 | 8.50 | | 9.03 | | 8.57 | 0.29 | |
|  | March 2015 | 7.86 | 8.48 | | 8.67 | | 8.36 | 0.25 | |
|  | June 2015 | 7.87 | 8.04 | | 8.26 | | 8.07 | 0.11 | |
|  | August 2015 | 8.63 | 8.77 | | 9.06 | | 8.77 | 0.11 | |

(a)

(b)

(c)
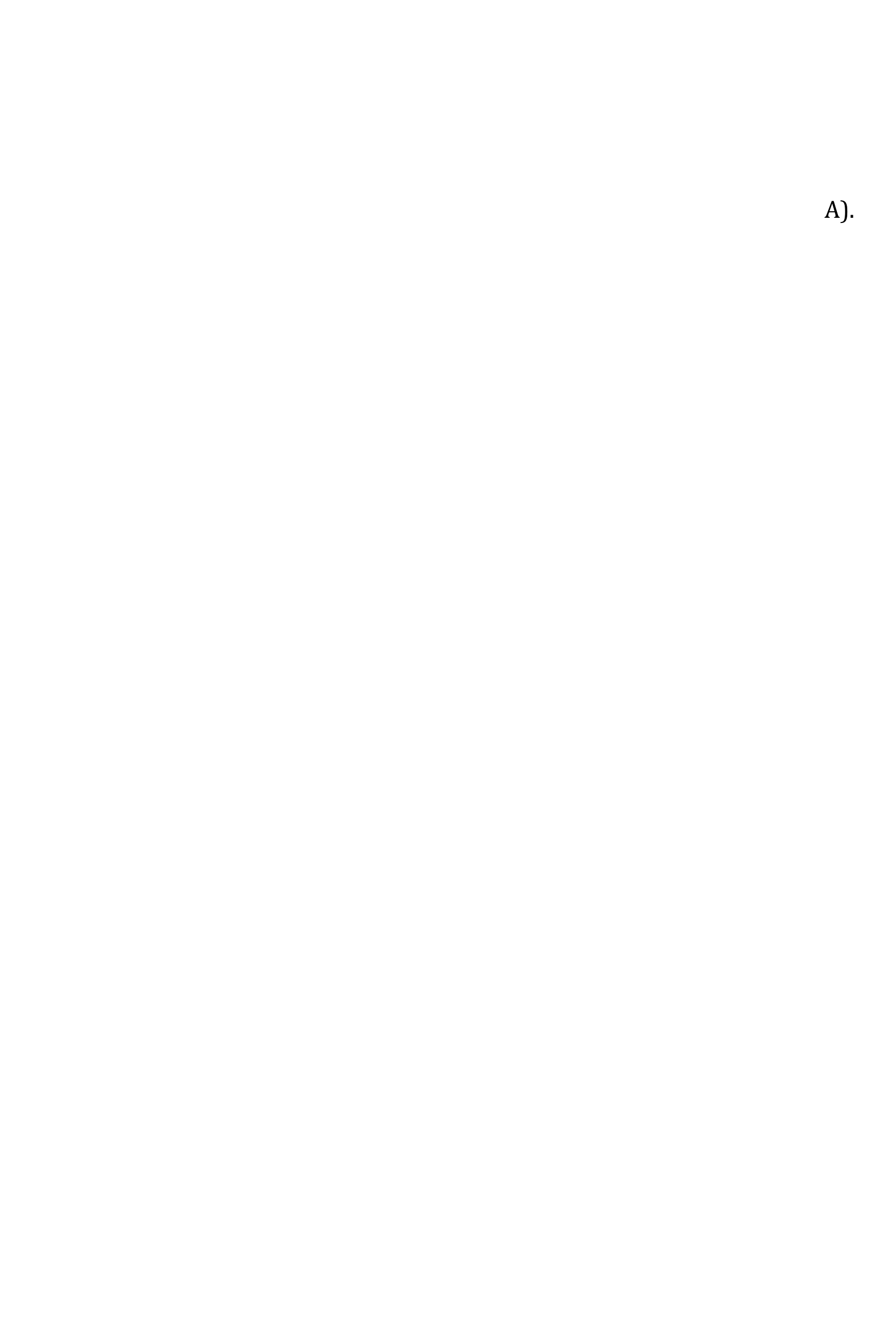
).

(d)

(e)

(f)

**Figure S1.** Nutrient concentrations (a-e, mg L^-1^) (f, ppt) by study riffle and time (Olentangy River, Ohio): (a) Total N (*F* = 262.95, *p* < 0.0001), (b) NO_3_ (*F* = 213.80, *p* < 0.0001), (c) Total P (*F* = 154.57, *p* < 0.0001), (d) PO_4_ (*F* = 13.90, *p* = <0.0001), (e) NH_4_ + PO_4_ (*F* = 1.25, *p* = 0.311), and (f) Total dissolved solids (*F* = 95.81, *p* < 0.0001). Significant pairwise differences are indicated by different letters a, b, c, d (Tukey’s HSD: *p* **<** 0.05).


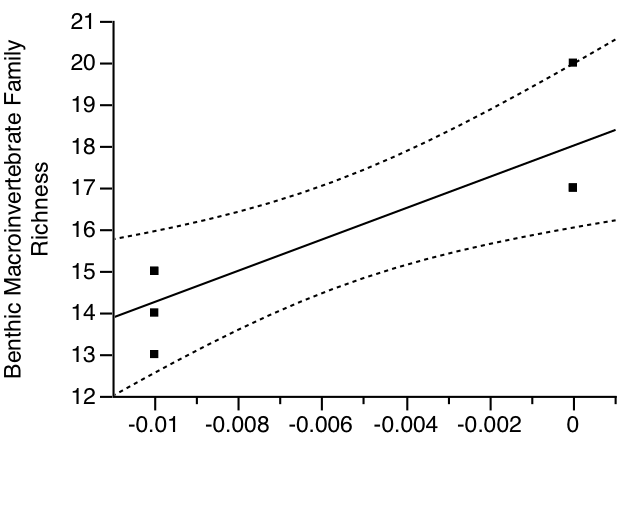


Two stacked data points

↵

Two stacked data points

↵

(a)

(b)


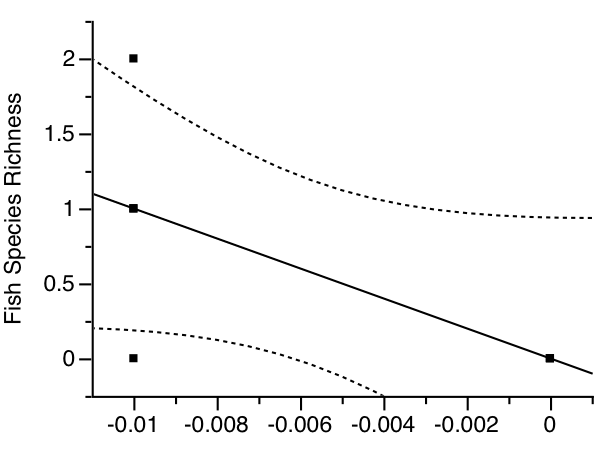


Δ Slope (m m^-1^)

Two stacked data points

↵

Three stacked data points

↵


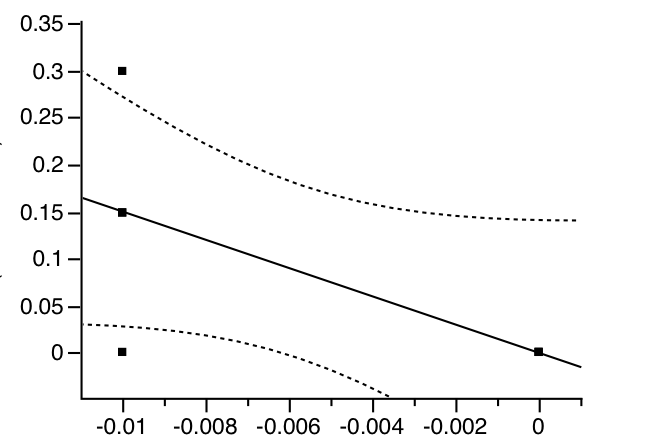


Fish Density (no. 2.25 m^-2^)

Three stacked data points

↵

Two stacked data points

↵

(c)

Δ Slope (m m^-1^)

**Figure S2.** Relationships between change in streambed slope between June 2014 and June 2015 (Δ Slope) and (a) benthic macroinvertebrate family richness (*y* = 18.0 + 375.0x*; R*^2^ = 0.73, *p* = 0.014), (b) fish species richness (*y* = 0.0 – 100.0*x*; *R*^2^ = 0.46 *p* = 0.093), and (c) fish density (*y* = -2.78e-17 -15.0*x*; *R*^2^ = 0.46, *p* = 0.093) across all study riffles. Dashed lines represent confidence curves at α = 0.05.

**Figure S3.** Benthic macroinvertebrate density pooled across time (no. 0.1 m^-2^) across study riffles in Olentangy River, Ohio (*F*_6,14_ = 6.25, *p* = 0.002). Significant pairwise differences based are indicated by different letters a, b (Tukey’s HSD: *p* **<** 0.05). Error bars are ±1 SE from the mean.

Macroinvertebrate Abundance

Year

**Figure S4.** Benthic macroinvertebrate abundance 1987-2015 (Ohio EPA 1999, 2005; Mike Bolton, OEPA personal communication). Note that these data were collected with Hester-Dendy samplers over > 6-week sample periods, and thus the abundances are much higher than that densities (collected with a Surber sampler for 90-s intervals) reported as study results in the main manuscript. In general, increases in abundance over time are attributed to improved water quality in the region.

(a)

Macro.Total

Riffle 1, 2014-11-01

Riffle 1, 2015-08-01

(b)

(c)

(d)

(e)

(f)

(g)

**Figure S5.** Redundancy analysis of benthic macroinvertebrate family richness (Macro.S) and density (Macro.Total) at each study riffle through time: (a) Riffle 1, (b) Riffle 2, (c) Riffle 3, (d) Riffle 4, (e) Riffle 5, (f) Riffle 6, and (g) Riffle 7. Blue arrows indicate how environmental variables were ordinated.

**References**

Ohio EPA. (1999). Biological and water quality study of the Olentangy River and selected tributaries 1999 – Delaware and Franklin Counties. OEPA Technical Report MAS/2000-12-6. State of Ohio Environmental Protection Agency, Division of Surface Water. Columbus, Ohio.

Ohio EPA. (2005). Biological and water quality study of the Olentangy River, Whetstone Creek and Select Tributaries, 2003-2004 – Crawford, Delaware, Franklin, Marion, and Morrow Counties. Ohio EPA Technical Report EAS/2005-12-6. State of Ohio Environmental Protection Agency, Division of Surface Water. Columbus, Ohio.
